# Balloon Venoplasty of Subclavian Vein and Brachiocephalic Junction to Enable Left Ventricular Lead Placement for Cardiac Resynchronisation Therapy

**DOI:** 10.1016/s0972-6292(16)30692-1

**Published:** 2013-11-15

**Authors:** Thanh Trung Phan, Simon James, Andrew Turley

**Affiliations:** James Cook University Hospital, Middlesbrough, UK

**Keywords:** Balloon Venoplasty of Subclavian Vein, Cardiac Resynchronisation Therapy

## Abstract

This report describes the successful implantation of a LV lead using balloon venoplasty to overcome a very tight stenosis of the right subclavian vein / brachiocephalic junction for cardiac resynchronisation therapy (CRT-P) in a patient with a right sided CRT-P system and a failed epicardial LV lead. It is important for device implanters to be familiar with interventional equipments and techniques such as balloon venoplasty to overcome difficult venous access.

## Introduction

Cardiac resynchronization therapy (CRT) has been shown to reduce morbidity and mortality in symptomatic patients with severe left ventricular systolic dysfunction (LVSD) and broad QRS duration [1]. There is an expanding population of patients with existing pacemakers who might require upgrade procedures to either CRT-P/D. Patients with pre-existing leads technically can pose several problems including central venous access and localised venous stenosis rendering subsequent lead placement difficult. Indeed, venous thrombosis is found in 35-40% of patients with transvenous pacing leads although complete occlusion is rare. [2]

This report describes the successful implantation of a LV lead using balloon venoplasty to overcome a very tight stenosis of the right subclavian vein / brachiocephalic junction for cardiac resynchronisation therapy (CRT-P) in a patient with a right sided CRT-P system and a failed epicardial LV lead.

## Case report

A 73 year old woman had a left-sided dual chamber pacemaker implantation for complete heart block following a mechanical aortic valve replacement (AVR) in 1996. In 2009 the patient's pacing system became infected and was extracted (generator - Guidant INSIGNIA, leads - PACESETTER TENRIL (Atrial) and CPI (RV)) and replaced with a right sided CRT-P system (Medtronic Syncra generator, RV lead-capSureFix Novus) in 2011 for symptomatic heart failure. There was difficulty accessing a suitable LV vein percutaneously and so she had an epicardial LV lead surgically implanted (Enpath Medical). Unfortunately this procedure was complicated by post-operative wound infection and a discharging sinus at the site of one of the video-assisted thoracic surgery (VATS) port incision wound but eventually made a good recovery. She had a severely impaired left ventricular systolic function and a paced ECG tracing (QRS=145ms) pre-CRT. She was on the following medications: bisoporol fumarate, enalapril, spironolactone, furosemide, simvastatin and warfarin.

**Figure 1:**
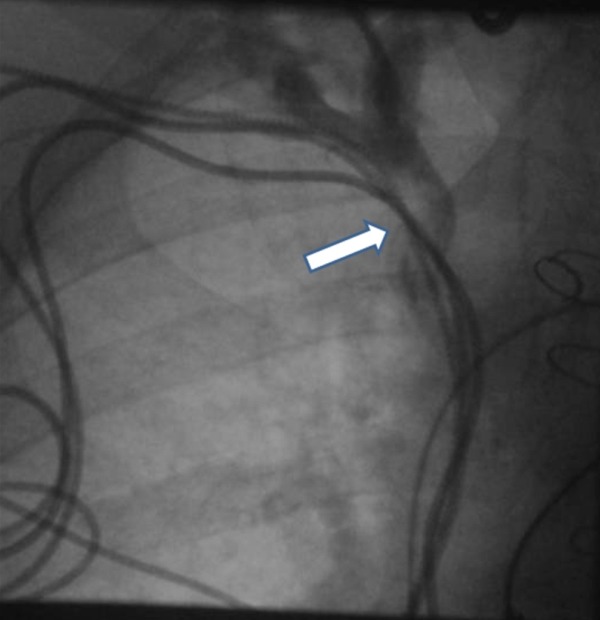
Venogram demonstrating severe obstruction of the left SVC and subclavian vein

The epicardial lead threshold gradually increased over a course of a year, from satisfactory level to 5.0V and then 8.0 V (0.9ms), with 97% ventricular pacing. The risks of epicardial lead extraction and re-position was felt to be unacceptably high and therefore the best way forward was to abandon the existing epicardial lead and for a new LV lead implanted transvenously (Medtronic Attain Starfix 4195). A peripheral venogram demonstrated a completely occluded left subclavian vein with collaterals to left internal jugular vein. In addition, she had a tight stenosis of the right subclavian vein secondary to pacing leads (see Figure 1). Clinically the patient had significant symptomatic benefit with CRT and therefore agreed to undergo peripheral balloon venoplasty to her right subclavian vein / brachiocephalic junction in an attempt to facilitate transvenous LV lead placement.

**Figure 2:**
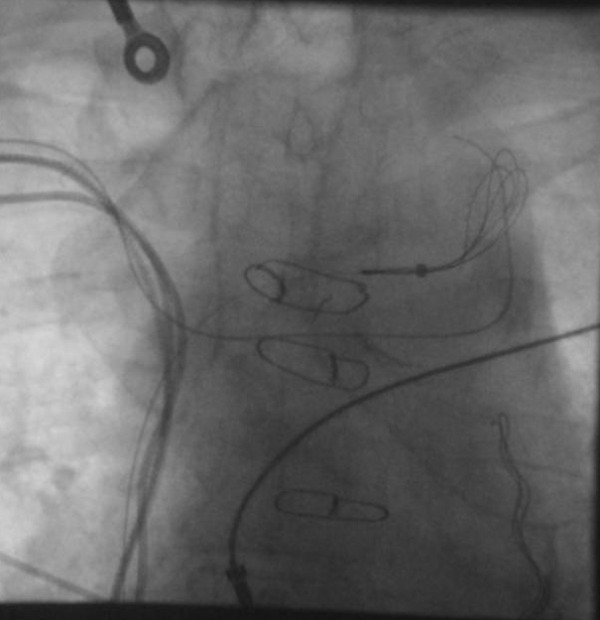
Snaring of the guide wire from the right subclavian vein

The procedure was performed under local anaesthetic with conscious sedation (Midazolam/Fenatnyl) by 2 operators. The right prepectoral system was dissected free and one subclavian vein (SC) puncture was performed under fluoroscopy guidance. Femoral venous and arterial access was also gained. A 0.35 wire was advanced to the point of occlusion. A 5Fr dilator was introduced over the wire and the 0.35 wire exchanged for a terumo wire. The terumo wire was fed down the right subclavian vein to the superior vena cava (SVC) and across to the left brachiocephalic vein. It was not possible to advance the terumo wire down the SVC into the right atrium nor was it possible to advance a PCI wire. A MPA2 catheter was passed to the left brachiocephalic vein from the right femoral vein which was then exchanged for an En-Snare over a 0.35 wire (see Figure 2) as access to right subclavian was not possible from the femoral veins.

**Figure 3:**
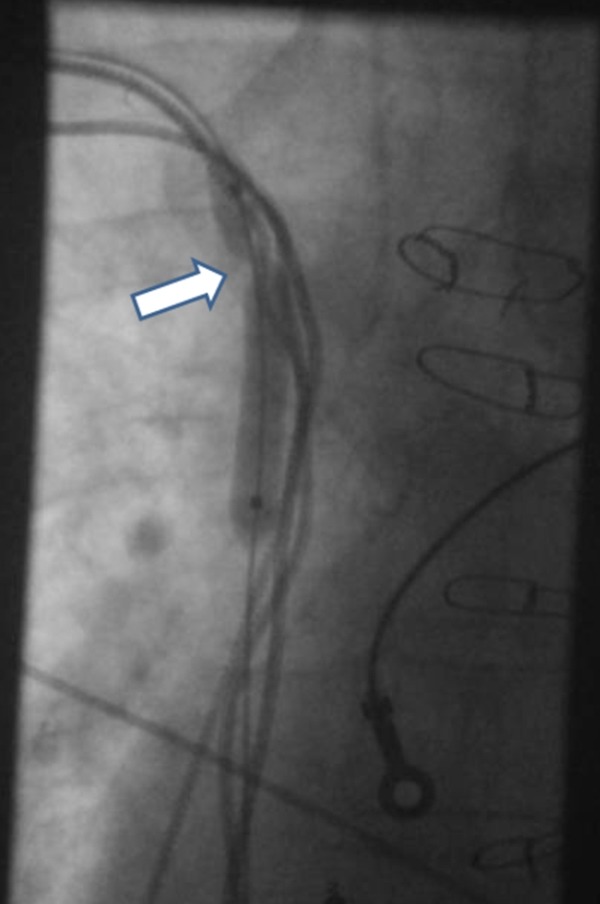
Balloon Venoplasty of the tight SVC stenosis

The terumo wire was then snared and pulled down to the inferior vena cava (IVC). A 4Fr Vert-Slip-Cath Beacon Tip (Cook Medical) was then passed over the terumo wire with ease down into the IVC. The terumo wire was exchanged for a 0.35 Amplatz super stiff wire and then snared from right femoral vein to providing extra support. A 6x40mm balloon (Powerflex P3 Cordis medical) was passed over the 0.35 wire and forwards from the distal SC to SVC where upon venoplasty was performed with a burst pressure of 15 atm. 30 seconds inflations were performed from SVC/RA junction all the way back to the original venous puncture site (see Figure 3).

**Figure 4:**
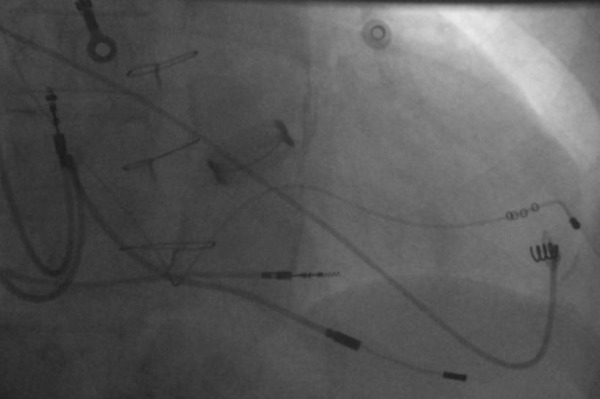
Successful placement of an Attain Starfix LV lead in the posterior-lateral cardiac vein

A Medtronic MB2 coronary sinus guide catheter was used to cannulate the CS. An Attain sub-selective catheter cannulated a poster-lateral branch of the coronary sinus which was double wired (choice floppy and choice floppy extra support). An Attain Starfix 4195 88cm LV lead was placed in the postero-lateral LV vein (Figure 4). The LV pacing threshold was 2.1V at 0.5ms with impedance of 1017Ω. The procedure was successful and took 150 minutes with no complications and patient was discharged the following day after satisfactory pacing checks and chest X-ray. At follow-up the patient reports significant improvement in symptoms and pacing parameters remain stable.

## Conclusion

Some degree of venous obstruction has been reported in almost 15% of patients prior to device implantation and can rise to 50% after transvenous device implantation in selected series. [3] Venous stenoses are often associated with the presence of multiple pacemaker/ ICD leads, history of venous thrombosis, use of temporary wire before implantation, use of hormone therapy, and temporary venous access for haemodialysis. These stenoses are often fibrotic in nature with early endothelisation of pacing leads and excessive proliferation of connective tissue leading to reduction in vessel lumen size. [2] With CRT-P/D indications continuously expanding, it important that device implanters become familiar with interventional equipments and techniques such as balloon venoplasty to overcome difficult venous access which has been demonstrated in this report and in others to be highly effective and safe. [4]
